# A recent distemper virus outbreak in the growing canine populations of Galapagos Islands: a persistent threat for the endangered Galapagos Sea Lion

**DOI:** 10.3389/fvets.2023.1154625

**Published:** 2023-05-09

**Authors:** Patricio Vega-Mariño, Jessie Olson, Ben Howitt, Rita Criollo, Lissette Figueroa, Solon Alberto Orlando, Marilyn Cruz, Miguel Angel Garcia-Bereguiain

**Affiliations:** ^1^Agencia de Regulación y Control de la Bioseguridad y Cuarentena para Galápagos, Santa Cruz, Galápagos, Ecuador; ^2^Programa de Doctorado de Salud Pública Humana y Animal, Universidad de Extremadura, Cáceres, Spain; ^3^Pan Animalia Galapagos, Santa Cruz, Galápagos, Ecuador; ^4^Worldwide Veterinary Service (WVS), Cranborne, United Kingdom; ^5^Universidad Espiritu Santo, Guayaquil, Ecuador; ^6^Instituto Nacional de Salud Pública e Investigación, Guayaquil, Ecuador; ^7^One Health Research Group, Universidad de Las Americas, Quito, Ecuador

**Keywords:** Canine Distemper Virus, Galapagos Islands, dogs, sea lions, distemper virus

## Abstract

Canine Distemper Virus (CDV) is a highly contagious virus that can cross mammalian species barriers and has widespread impacts on both domestic animals and wildlife populations. This study describes a recent outbreak of CDV in the Galapagos Islands in 2019. A total number of 125 dogs with clinical signs compatible with CDV were included in this study. Nasal swabs were taken and analyzed by RT-qPCR for the detection of CDV, resulting in a positivity rate of 74.4% (IC95%, 66–81%). Among the CDV positive dogs, 82.2% presented with respiratory signs, 48.8% neurological signs, and 28.9% gastrointestinal signs. CDV has been previously reported in the domestic canine population of the Galapagos Islands in 2001 and 2004. The current study shows how CDV is still a threat for the endemic and endangered Galapagos sea lion, despite recent policies for dog population control and CDV vaccination.

## Introduction

The Galapagos Islands are a World Heritage Site with one of the world's highest concentrations of endemic species (1) and it is an iconic location for evolutionary and conservation biology. Invasive species such as domestic canines and felines are still a threat to the biodiversity of the archipelago. In the most populated island of Santa Cruz alone, the population of domestic canines was estimated at 3,886 in 2018, representing a 55% increase from a previous census in 2014 (2). With domestic canine overpopulation, and the risk of its continued growth, comes the potential for habitat encroachment, predation of endemic wildlife, and interspecies transmission or spill over of infectious diseases, such as Canine Distemper Virus (CDV), into wildlife populations (3).

CDV is one of the leading causes of death in domestic canines in the world (4) and is most commonly spread through close contact with infected hosts *via* aerosol or through body secretions. Domestic dogs infected with CDV present with various clinical manifestations, and mortality rates that depend on the viraemic stage, host age, environmental stress and immune status of the host (4). CDV is multisystemic, leading to pathologies of the respiratory and gastrointestinal systems, as well as cutaneous tissues (4). CDV signs may also manifest as neurological abnormalities that vary in severity, which can occur between 1- and 3-weeks post infection. These neurological signs can be observed in conjunction with the previously mentioned clinical signs, or they may manifest after a subclinical infection (5).

CDV is highly prevalent worldwide (6), and prior to this study, the presence of CDV was reported in the canine population of the Galapgos on two occasions—in 2001 and in 2004 (3). In 2001, an outbreak of CDV killed more than 600 dogs in Santa Cruz and Isabela Islands (7). Also, 95 dogs were tested within a surveillance program on Isabela Island in 2004, showing a 22% seropositivity to CDV through ELISA testing (7).

CDV exhibits the ability to adapt to a broad number of hosts, making its eradication considerably difficult and of concern for cross species transmission. Epidemics caused by morbilliviruses, such as CDV, have been observed amongst different marine mammals (6), with seals and sea lions showing high susceptibility, as reported in Harbor seals in the Baltic Sea (8) and Caspian Seals (9). Though the route of transmission was unconfirmed, terrestrial carnivores were the suspected vectors for disease (9). CDV infection has also been reported in the endemic and endangered Galapagos Sea Lion (*Zalophus wollebaeki*), with six pups testing positive during a surveillance program carried out in 2011, although the source of exposure was not confirmed (10, 11).

Despite the growing canine population in Galapagos Islands, there has been no CDV surveillance for more than a decade, and therefore no current data available. This is particularly relevant because canine vaccination, including that against CDV, have only been conducted since 2017. The present work reports a recent outbreak of CDV amongst the domestic canine population of Santa Cruz Island in 2019.

## Materials and methods

### Data and sample collection

A total of 125 dog with signs of CDV on Santa Cruz Island during July to August 2019 were included in the study, after the veterinarians detected several cases of dogs with pathognomonic signs of infection. Data collection was circumstantial based on owner compliance and clinical severity of the case. Age, clinical signs, owners address, and disease history were obtained where possible and appropriate.

### Sample analysis using RT-PCR

All samples were processed at “Agencia de Regulación y Control para la Bioseguridad y Cuarentena de Galápagos” in Puerto Ayora, Santa Cruz, using RT-PCR testing. RT-PCR is considered a sensitive and accurate molecular diagnostic assay (5) able to detect RNA of the nucleoprotein gene (NP) in serum, blood, saliva and pharyngeal samples. RNA extraction was performed from nasal swab samples using the PureLink^®^ Viral RNA/DNA Mini Kit (Invitrogen, Life Technologies Corporation, Carlsbad, CA 92008 USA), following manufacturer instructions. RNA samples were stored at −20°C until used. The synthesis of cDNA was performed using the SuperScript^®^ III First-Strand Synthesis SuperMix for qRT-PCR (Invitrogen, Life Technologies Corporation) according to the manufacturer's recommendations. Quantification and verification of RNA integrity was performed using the Qubit^®^ 3.0 Fluorometer (Thermo Scientific, USA) following the manufacturer's protocol and then visualized in 1% agarose gel stained with ethidium bromide, using an Enduro™ GDS (Labnet) imaging screen. Internal primers were used to verify the stability of the synthesized cDNA.

Primers were selected from a previous publication (12), and they are detailed in Table 1. The mastermix contained: 12 μL DreamTaq Green MasterMix 2X (Thermo Fisher Scientific; active ingredients: 0.4 mM DreamTaq DNA Polymerase, 2X DreamTaq Green buffer, dATP, dCTP, dGTP, and dTTP; 4 mM MgCl_2_), 1 μL 10 μM forward first primer, 1 μL 10 μM reverse first primer, 9 μL sterile water. Template DNA (2 μL) was added to each well for a final volume of 25 μL.

**Table 1:** Primers sequences and PCR fragment sizes for CDV and GAPDH (nucleic acid extraction quality control).

**Primer**	**Sequence (5^′^-3^′^)**	**Target gene**	**Size (bp)**
*P1-F*	ACAGGATTGCTGAGGACCTAT	CDV-NP	287
P2-R	CAAGATAACCATGTACGGTGC		
*GAPDH p7*	GCCAAAAGGGTCATCATCTC	Control (dog DNA)	229
*GAPDH p8*	GGCCATCCACAGTCTTCT		

Thermal cycling conditions were as follows: 95°C for 4 min, 35 cycles [94°C for 45 s, 55°C for 45 s, 72°C for 45 s], 72°C for 7 min, hold at 4°C. The results were visualized using a 1.5% agarose gel (UltraPure™, Invitrogen™, Thermo Fisher Scientific) in TBE buffer (QIAGEN Sciences) with ethidium bromide (Promega Corporation, Madison, WI 53711, USA). Primers GAPDH p7 and GAPDH p8 were used under the same conditions as P1-F and P2-R.

## Results

### CDV positivity rate among dogs with signs compatible with CDV infection during the 2019 outbreak

A total of 125 dogs with signs of CDV infection were tested using RT-PCR during the outbreak. Of those, 93 tested positive and 32 tested negative, yielding a positivity rate of 74,4% (CI95%, 66–81%).

### Clinical observations in confirmed cases of CDV infection

Due to several variables including veterinarian volunteer turnover rates, there was inconsistent monitoring of the outbreak and collection of clinical histories. The clinical signs were recorded for 45 of the positive dogs and 12 of the negative dogs. The recorded clinical signs of the 45 CDV positive cases were categorized as gastrointestinal, respiratory, and neurological (Table 2). Each one of these categories can be further subdivided into more specific clinical signs. Within that of the respiratory signs, the two most common were coughing and ocular discharge. Involuntary muscular tremors were the most common signs of the neurological ones, and vomiting was the most common of the gastrointestinal signs. The most common signs observed in all CDV infected dogs were coughing, lethargy, and anorexia. Respiratory signs ranged from mild dry coughs to progressive wet honking coughs indistinguishable from canine kennel cough. However, these were also frequently observed in the CDV negative dogs. On the other hand, regarding more severe respiratory signs in CDV positive dogs, congestion of the lungs presented predominantly in the ventral lobes, and often associated with increased ocular and/or nasal discharge, ranging from clear and runny to mucopurulent secretions.

**Table 2:** Frequency of clinical signs seen amongst the 45 positive cases of CDV.

**Signs**	**Prevalence of each kind of signs (%)**	**Clinical signs**	**Number of cases (total 45)**	**Prevalence (%)**	**Confidence Interval (CI 95%)**	
					**Lower limit (%)**	**Upper limit (%)**
Gastrointestinal	28.9%	*Vomiting*	10	22.20	14.91	29.49
		*Diarrhea*	5	10.10	4.82	15.38
Respiratory	82.2%	*Coughing*	27	60.00	51.41	68.59
		*Sneezing*	6	13.30	7.35	19.25
		*Ocular discharge*	22	48.90	40.14	57.66
		*Nasal discharge*	18	40.00	31.41	48.59
Neurological	48.8%	*Tremors*	13	28.80	20.86	36.74
		*Spasms*	10	22.20	14.91	29.49
		*Seizures*	9	20.00	12.99	27.01
Non-specific signs		*Lethargy*	31	68.90	77.02	60.78
		*Anorexia*	26	57.70	66.36	49.04

The more severe cases of CDV infection presented with definitive neurological signs, often without any prior symptoms having arisen. The neurological signs were predominantly involuntary muscle spasms and tremors, particularly those of the masticatory muscles. Tremors of the temporal and masseter muscles were associated with increased jaw tone and variable cranial nerve deficits, particularly facial paralysis. Seizures were also common, from full-body seizures to “chewing gum” or “lip-smacking” fits. The most severe neurological signs observed were relentless full body tremors, associated with bilateral hind limb paresis that eventually progressed to quadriparesis. All quadriparesis cases showed drastically reduced spinal reflexes and some had inhibited deep pain responses. It should be mentioned that some CDV negative dogs also presented neurological signs compatible with CDV disease.

### Death and euthanasia observations

The number of dogs that died during this CDV outbreak was not determined due to the inability to continuously monitor all the infected animals. Many dogs with mild signs were often isolated at home in order to contain the viral spread and never returned to follow up with the veterinarians, so the death rate is likely to be higher than calculated.

As far as it was formally recorded, 20 (16%) of the 125 sampled cases died; 16 of them were euthanized for welfare reasons. 15 of the 20 dogs that died were positive for CDV. So, the mortality rate for the CDV was 16.1% (15/93).

### Spatial distribution within Santa Cruz Island

Using the addresses provided by owners, we could map CDV positive and negative dogs into respective rural and urban neighborhoods. 74.5% of CDV positive cases were found within the urban areas, and 25.5% within the rural areas of Santa Cruz Island.

## Discussion

Our study describes the outbreak of CDV in urban and rural canine populations in Santa Cruz Island during 2019. The CDV positivity rate among dogs having signs related to CDV was as high as 74%. For the CDV positive dogs, respiratory, neurological and gastrointestinal symptoms were most commonly observed.

Santa Cruz Island has experienced a domestic canine population growth over the last few years. The last survey of the domestic canine population in Santa Cruz Island was conducted in 2018, showing a dog population increase of 55% in comparison to a previous census in 2014. There was also an additional increase in households owning a dog from 40% in 2014 to 58% (2). A greater dog population density would facilitate CDV outbreaks and spread, with a higher number of interactions between hosts and other canines facilitating its exchange and dispersion within the population. CDV typically causes epidemics with a periodicity dependent on the build-up of susceptible individuals (13). The vaccination status of a population can contribute to this. It is worth mentioning that animal vaccines were historically banned in the Galapagos Islands, and this policy was only revised in 2017, when several dog vaccines were that included CDV. So, the current CDV vaccination covarage in Galapagos was not substantial enough to prevent this outbreak. CDV vaccination should be reinforced to potentially eradicate CDV from the Galapagos Islands, as long as the illegal trafficking of dogs to the islands is also controlled (3, 7).

The CDV outbreak described in this study affected both rural and urban populations. This is important to consider in terms of potential spill over to the iconic and endangered Galapagos sea lion *(Zalophus wollebaeki)* populations. CDV was found in dead pups of Galapagos sea lions from San Cristobal Island in 2011–2012 (10, 11). This is significant in the coastal urbanized area of Puerto Ayora, where the common presence of Sealions results in a high number of dog-sealion interactions and presents a high risk of CDV transmission. The presence of CDV within the rural populations is also worrying, as free-roaming populations give rise to reservoirs of the disease, presenting a challenge to CDV eradication.

Our study has some limitations associated with budgetary restrictions. No serological test was carried out from the collected blood samples to confirm the seroconversion of the RT-PCR positive dogs. Also, no further characterization of the CDV positive samples by genome sequencing was carried out; this is particularly important to determine if the outbreak was caused by one single CDV strain. Nevertheless, although illegal trafficking of dogs to the island cannot be ruled out, the level of isolation of Santa Cruz island would make a single strain of CDV the most plausible explanation behind this outbreak. As a future direction of this study, CDV will be continued to be monitored as well as the further characterization of future samples described. This will be particularly interesting to evaluate the success of future CDV vaccination campaigns in the Galapagos Islands, as vaccination amongst this dog population is novel and unstudied.

In conclusion, the risk of CDV outbreaks in the Galapagos Islands persists and represents a risk for the iconic and endangered Galapagos sea lion. The enforcement of animal movement restrictions from the continent and between islands, the implementation of regular CDV vaccination campaigns, and the mandatory neutering of animals are all potential steps to eventually eradicate CDV in Galapagos.

## Data availability statement

The original contributions presented in the study are included in the article/supplementary material, further inquiries can be directed to the corresponding authors.

## Ethics statement

This study was authorized and carried out under the leadership of Agencia de Regulación y Control para la Bioseguridad y Cuarentena de Galápagos, the Ecuadorian Governmental Agency responsible for domestic animal health in Galapagos Islands. According to the Ecuadorian regulations for animal research, sample collection from domestic animals for disease diagnosis and surveillance does not required IRB approval. Sample collection was carried out by certified veterinarians. Written informed consent was obtained from the owners for the participation of their animals in this study.

## Author contributions

All authors listed have made a substantial, direct, and intellectual contribution to the work and approved it for publication.
